# The contribution of bullying victimisation to the burden of anxiety and depressive disorders in Australia

**DOI:** 10.1017/S2045796019000489

**Published:** 2019-09-19

**Authors:** Amarzaya Jadambaa, Hannah J. Thomas, James G. Scott, Nicholas Graves, David Brain, Rosana Pacella

**Affiliations:** 1 Australian Centre for Health Services Innovation, Institute of Health and Biomedical Innovation, Queensland University of Technology Kelvin Grove, Brisbane, QLD 4059, Australia; 2 School of Public Health and Social Work, Queensland University of Technology Kelvin Grove, Brisbane, QLD 4059, Australia; 3 Queensland Centre for Mental Health Research, The Park Centre for Mental Health, Wacol, QLD 4076, Australia; 4 Faculty of Medicine, Centre for Clinical Research, The University of Queensland, Herston, QLD 4029, Australia; 5 Faculty of Medicine, School of Public Health, The University of Queensland, Herston, QLD 4006, Australia; 6 Metro North Mental Health, Royal Brisbane and Women's Hospital, Herston, QLD 4029, Australia; 7 Research Office, University of Chichester, West Sussex, UK

**Keywords:** Behaviour problems, health outcomes, mental health, risk factors

## Abstract

**Aim:**

There is now a strong body of literature showing that bullying victimisation during childhood and adolescence precedes the later development of anxiety and depressive disorders. This study aimed to quantify the burden of anxiety and depressive disorders attributable to experiences of bullying victimisation for the Australian population.

**Methods:**

This study updated a previous systematic review summarising the longitudinal association between bullying victimisation and anxiety and depressive disorders. Estimates from eligible studies published from inception until 18 August 2018 were included and meta-analyses were based on quality-effects models. Pooled relative risks were combined with a contemporary prevalence estimate for bullying victimisation for Australia in order to calculate population attributable fractions (PAFs) for the two mental disorder outcomes. PAFs were then applied to estimates of the burden of anxiety and depressive disorders in Australia expressed as disability-adjusted life years (DALYs).

**Results:**

The findings from this study suggest 7.8% of the burden of anxiety disorders and 10.8% of the burden of depressive disorders are attributable to bullying victimisation in Australia. An estimated 30 656 DALYs or 0.52% (95% uncertainty interval 0.33–0.72%) of all DALYs in both sexes and all ages in Australia were attributable to experiences of bullying victimisation in childhood or adolescence.

**Conclusion:**

There is convincing evidence to demonstrate a causal relationship between bullying victimisation and mental disorders. This study showed that bullying victimisation contributes a significant proportion of the burden of anxiety and depressive disorders. The investment and implementation of evidence-based intervention programmes that reduce bullying victimisation in schools could reduce the burden of disease arising from common mental disorders and improve the health of Australians.

## Introduction

Bullying during childhood and adolescence is a significant public health issue in Australia. Contemporary prevalence estimates indicate that approximately 15% of children and adolescents (at least one in seven) have experienced bullying victimisation within the previous 12 months (Thomas *et al*., 2017; Jadambaa *et al*., 2019). Bullying by definition is a negative action on the part of one or more individuals that includes three components: intention to harm, repetition and a power imbalance between a victim and the perpetrator(s) (Olweus, 1993; Olweus, 2013). There is now a strong body of evidence that suggests experiences of bullying victimisation (*being bullied*) precedes the later development of mental illness (Moore *et al*., 2014; Moore *et al*., 2017). The negative consequences of bullying victimisation are not limited to childhood and adolescence and can persist into adulthood. Victims have been consistently found to be at an increased risk of internalising problems, in particular diagnoses of later anxiety and depressive disorders in adulthood (Hemphill *et al*., 2011; Copeland *et al*., 2013; Stapinski *et al*., 2014; Takizawa *et al*., 2014). Not only is bullying victimisation associated with an increased risk of these common mental disorders, but it also results in substantial costs for individuals, their families and society at large (Wolke and Lereya, 2015; Moore *et al*., 2015*b*).

Researchers have undertaken systematic reviews and meta-analyses examining the association between bullying victimisation and a range of health outcomes. Ttofi *et al*. (2011) conducted the first systematic review and meta-analysis of longitudinal studies and concluded that children who were bullied at school were twice as likely to develop depression compared to those who had not experienced bullying. This study focused on the later development of depression only. Another systematic review and meta-analysis (studies from inception until February 2015) identified mental disorders and substance use as the main consequences of bullying victimisation (Moore *et al*., 2017). This analysis summarised the cross-sectional as well as longitudinal evidence separately in order to examine the dimension of time. The review concluded there was convincing evidence for a causal relationship between bullying victimisation and anxiety and depressive disorders in particular.

According to the most recent national survey, approximately one in five Australians aged 16–85 years meet the criteria for a mental disorder in the previous 12 months, which is the equivalent of 3.2 million Australians (Slade *et al*., 2009). Overall, anxiety and depressive disorders (14.7 and 6.2%, respectively) were among the most commonly diagnosed (Slade *et al*., 2009). The most recent Global Burden of Disease Study (GBD 2017) estimated that mental disorders ranked sixth in terms of overall disability-adjusted life years (DALYs) globally, and ranked fourth in Australia. Within the mental disorders group, depressive disorders (major depressive disorder and dysthymia) followed by anxiety disorders accounted for the most DALYs in Australia (Kyu *et al*., 2018).

In GBD 2017, the burden of disease attributable to bullying victimisation was assessed for the first time. Overall, 0.16% of total DALYs for all disease causes for both sexes and all ages in Australia were attributable to bullying victimisation (Stanaway *et al*., 2018). When the estimates were further disaggregated by age group and disease cause, 12.2% of total DALYs for anxiety disorders, and 9.7% of total DALYs for depressive disorders were attributable to bullying victimisation for both sexes within the age group 10–24 years in Australia (Stanaway *et al*., 2018). The methodology used in global studies is often not well described limiting reproducibility (AbouZahr *et al*., 2017). As a result, there is a need for a local study to provide understanding of the Australian context to inform policy decisions. The current study sought to better understand how bullying victimisation among Australians influences the burden of the most common mental disorders, anxiety and depression. This study can support priority-setting and resource allocation decisions in the local context. The estimates from this study are the first comparison with those reported in GBD 2017.

The first aim of this study was to summarise the longitudinal evidence of an association between bullying victimisation and the later development of anxiety and depressive disorders. The second aim of this study was to estimate the burden of anxiety and depressive disorders attributable to child and adolescent bullying victimisation in Australia, based on the 12-month point prevalence estimated in a previous systematic review and meta-analytic study (Jadambaa *et al*., 2019).

## Methods

Exposure to bullying victimisation was treated as a risk factor for anxiety and depressive disorders, using counterfactual estimation and comparative risk assessment methods (Stanaway *et al*., 2018). This involved comparing the current local health status with the theoretical minimum risk exposure level assumed to be zero exposure to bullying victimisation. Population attributable fractions (PAFs) were determined by the prevalence of exposure to bullying victimisation in the Australian population and the relative risks (RRs) of disease occurrence given exposure. This methodology has been used to estimate the burden of a related form of interpersonal violence, exposure to child maltreatment (Moore *et al*., 2015*a*).

### Types of bullying victimisation

Traditional bullying typically occurs face-to-face, and cyber bullying occurs in an online environment (Smith *et al*., 2008). Exposure to bullying victimisation was included in this study where individuals are exposed to bullying in childhood and adolescence as victims only (*being bullied* – bullying victimisation) or as victim-perpetrators (*both being bullied and bullying others* – bullying victim-perpetration). Experiences of perpetrators (bullying others – bullying perpetration) were excluded.

### Prevalence of exposure

Prevalence estimates from another systematic review and meta-analysis were used (Jadambaa *et al*., 2019). This study estimated the 12-month prevalence of self-reported bullying victimisation experienced among Australian children and adolescents at 15.17%. This estimate included prevalence data for traditional as well as cyber forms of bullying victimisation (Table 1).

**Table 1.** Results of meta-analysis of the prevalence of bullying victimisation in childhood and adolescence in Australia (Jadambaa *et al*., 2019)

Type of Involvement	Recall period	Data points	Pooled prevalence %	95%CI	*I*^2^ (%)	Cochran's *Q*	Test for heterogeneity (*p*-value)
Bullying victimisation exposure^a^^,^^b^	12 months	35	15.17	9.17–22.30	99.65	9804.70	<0.001

a Where studies reported victimisation only and victim-perpetration estimates, they were combined to give an overall victimisation rate that would be comparable to studies that did not specify the victim-perpetration grouping.

b Where studies reported traditional bullying, cyber bullying, traditional and cyber bullying (included both estimates), and not specified whether cyber or traditional bullying, they were combined to give an overall estimate.

### Mental disorders

In this study, mental disorders were classified according to the categories specified by the Diagnostic and Statistical Manual of Mental Disorders (DSM-IV-TR) (APA, 2000) and the International Classification of Diseases 10 (WHO, 1992), which align with the diagnostic tools reported in published cohort studies. *Anxiety disorders* included generalised anxiety disorder, agoraphobia and panic disorder, and social phobia, specific phobia and anxiety disorders not otherwise specified. *Depressive disorders* included major depressive disorder and dysthymia.

### Relative risk estimates

#### Search strategy

This study updated a previous systematic review and meta-analysis (Moore *et al*., 2017) which reported studies identified from inception to January 2015. The processing and reporting of results are based on the recommendations from the Preferred Reporting Items for Systematic Reviews and Meta-Analyses (PRISMA) (Moher *et al*., 2010). The complete PRISMA checklist is presented in Appendix 1. The systematic search identified cohort studies that examined the association between bullying victimisation during childhood/adolescence and the later development of anxiety and depressive disorders. A review protocol was developed with search methods and inclusion/exclusion criteria specified in advance (Appendix 2). Four electronic databases (PubMed, EMBASE, ERIC and PsycINFO) were searched between 1 January 2015 and 18 August 2018 using the terms: ‘child*’, adolescen*, ‘bull*’, ‘victim*’, ‘harass*’, ‘outcome’, ‘anxiety’, ‘depress*’, ‘longitudinal’, ‘cohort’, ‘Jan 2015–Aug 2018’. In addition, reference lists of included studies were screened for any other relevant study and authors were contacted to obtain more detailed information, as needed. Articles in languages other than English were translated if they were deemed relevant.

#### Inclusion and exclusion criteria

This systematic review included studies meeting the following inclusion criteria: (1) published in a peer-reviewed journal, (2) examined an association between exposure to bullying victimisation as a child or adolescent and later development of anxiety and depressive disorders, and (3) the study was longitudinal and population-based. Some studies reported associations for victimisation as well as victim-perpetration; in these cases, both estimates were included. Where available, the unadjusted and adjusted odds ratios (ORs) for bullying victimisation including victim-perpetration for anxiety and depressive disorders were extracted separately. Included studies reported effect sizes and 95% confidence intervals (CIs) comparing those exposed and not exposed. Alternatively, included studies provided the information from which effect sizes and CIs could be calculated. In the few instances where the same sample was reported across different publications, the most informative article was selected: for example, studies reporting sex- or age-specific prevalence estimates were selected over those providing combined estimates. All longitudinal cohort studies previously included by Moore *et al*. (2017) were also assessed against inclusion and exclusion criteria.

#### Data extraction and synthesis

The full text of papers that met inclusion criteria was retrieved and examined. The first author (AJ) independently assessed the articles for eligibility and any uncertainties were resolved through discussion with HT and RP. The following details were extracted for each study: study design, country, sample size, gender, follow-up period, assessment of bullying victimisation and health outcomes (Appendix 3).

There was a significant variation across studies in terms of model adjustments, which meant it was necessary to further explore the effects of adjustment over a series of sub-group analyses. Some studies controlled for demographics only (e.g. gender and age), environmental and/or family factors only (e.g. having a friend and parental social class) or outcomes at baseline only (e.g. anxiety or depression), whereas others controlled for a combination of variables. Also, a few studies reported unadjusted effect sizes. In order to account for different adjustment methods, the extracted data points were grouped so they were analysed in three sub-group analyses: (i) unadjusted, (ii) adjusted for demographic, family and/or environmental factors and (iii) adjusted for mental health outcomes at baseline in addition to demographic, family and/or environmental factors (Table A2, Appendix 3). Similarly, separate subgroup analyses were conducted for victimisation only and victimisation including victim-perpetration.

#### Quality assessment

Quality of studies was assessed using an adapted version of the Newcastle–Ottawa Scale for cohort studies (Wells *et al*., 2000). This tool has been used in a previous systematic review and meta-analysis and described in more detail in Appendix 2 (Norman *et al*., 2012). The quality assessment for each study is presented in Appendix 3. The total quality score for each study was the sum of the scores for individual assessment items. This was converted to a proportional quality score (the total quality score divided by 11, which was the maximum score possible) for use in a tool for meta-analysis in Microsoft Excel namely Meta-XL version 5.3.

#### Statistical analyses

*Relative risk estimates and meta-analyses*. Weighted summary measures were computed using MetaXL version 5.3, a plugin package for Microsoft Excel (Barendregt *et al*., 2013). RRs were chosen as the principal summary measure. If ORs were not reported in included studies, ORs and their 95% CIs were calculated based on provided exposed/non-exposed case numbers and exposed/non-exposed non-case numbers using a cohort study OR calculator in STATA 15.0 (StataCorp, 2017). All ORs were then converted to RR estimates using an imputation method which reconstructs fourfold tables and event frequency values from published and estimated ORs and their 95% CIs, given the sample sizes (Di Pietrantonj, 2006). The meta-analyses were then carried out using reconstructed RR estimates. In some cases, it was necessary to use reported ORs as an approximation of RR when there was insufficient information to do the OR-to-RR conversion (Davies *et al*., 1998). Specifically, four studies did not report the prevalence of depressive/anxiety disorders in the non-exposed group, and in these instances, the OR = RR assumption was made. Models were later tested with and without these four studies included to ensure there were no significant differences in the RR estimates.

A quality effects meta-analytic model was used to pool the RR estimates. This is a modified version of the fixed-effects inverse variance method that allows giving greater weight to studies of high quality and lower weight to studies of lesser quality by using the quality scores assigned to each study (Doi and Thalib, 2008; Doi *et al*., 2011). Heterogeneity was quantitatively assessed using the Cochran's *Q* and *I*^2^ statistics to evaluate whether the pooled studies represent a homogeneous distribution of effect sizes. Evidence of publication bias was investigated by means of funnel plots using the standard error on the *y*-axis.

*Calculation of PAFs and attributable burden*. The estimated pooled RRs calculated for anxiety and depressive disorders which were adjusted for key cofounders including the presence of mental disorders at baseline were paired with the prevalence estimate for bullying victimisation (Jadambaa *et al*., 2019) to calculate PAFs using the following formula (Levin, 1953):

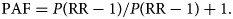


In this formula, ‘*P*’ is the prevalence of bullying victimisation and ‘RR’ is the relative risk of anxiety and depressive disorders from meta-analyses adjusted for demographic, environmental and family factors as well as anxiety and depression at baseline. PAFs were then applied to estimates of the burden of disease in Australia from GBD 2017 (Kyu *et al*., 2018) for anxiety and depressive disorders, measured in DALYs [DALY  =  years of life lost due to premature death (YLL) + years lived with disability (YLD)].

*Uncertainty analysis*. Macro simulation-modelling techniques and MS EXCEL software were used to calculate uncertainty ranges around pooled point estimates. This interval reflects the main sources of sampling uncertainty in the calculations used (uncertainty in the prevalence of exposure and RRs).

## Results

### Systematic review, meta-analysis and relative risk estimates for bullying victimisation and health outcomes

**Fig. 1.**
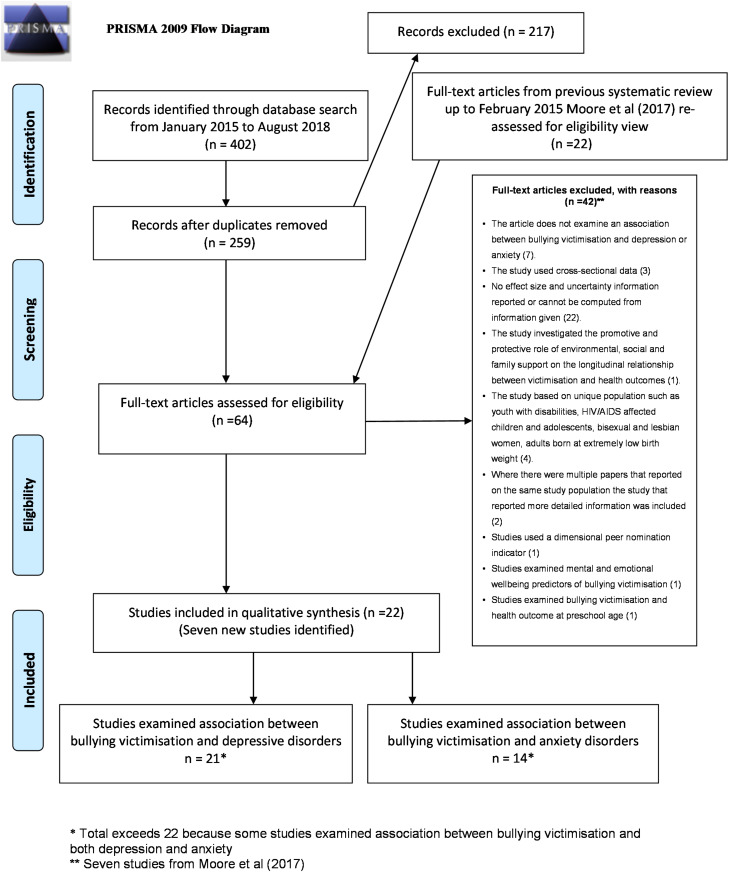
PRISMA flow diagram showing the process of study selection for inclusion in systematic review. *Total exceeds 22 because some studies examined association between bullying victimisation and both depression and anxiety. **Seven studies from Moore *et al*. (2017).

**Fig. 2.**
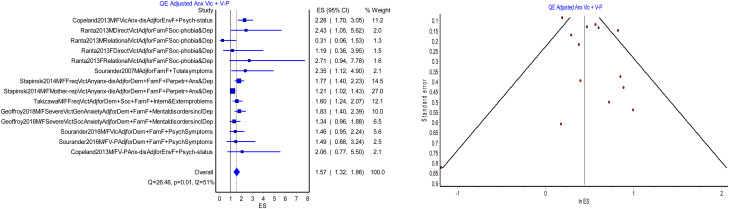
Relationship between bullying victimisation and anxiety disorders (adjusted for baseline anxiety). Individual and combined relative risks.

**Fig. 3.**
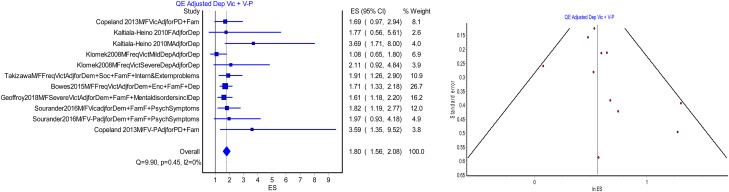
Relationship between bullying victimisation and depressive disorders (adjusted for baseline depression). Individual and combined relative risks.

A total of 402 articles were identified by the electronic database search, of which 143 were duplicates. Titles and abstracts for 259 unduplicated references were reviewed and a further 217 articles were excluded. Of the 64 studies assessed for eligibility, 22 longitudinal studies satisfied the pre-determined inclusion criteria [including 15 studies from the original published systematic review (Moore *et al*., 2017), and seven newly identified studies] (Fig. 1, Appendix 4). Length of follow-up time ranged from 6 months to 34 years. Studies were all conducted in high-income regions consisting of Europe (*N*  =  12), North America (*N*  =  7) and Australia (*N*  =  3). Some studies examined the association between bullying victimisation and both depressive and anxiety disorders, while others examined the association between bullying victimisation and anxiety disorders only or depressive disorders only. Characteristics for all included studies are summarised in Appendix 3 (Table A1), along with the quality assessment procedure (Wells *et al.*, 2000) and the total quality score for each study (Appendix 3, Table A2). Scores ranged from 4.5 to 10 out of 11. The test for heterogeneity was highly significant, with *p* < 0.001 for all groups. Forest plots and funnel plots to visualise individual analyses as well as pooled estimates are presented in Appendix 4 (Figs 2, 3).

The results of the meta-analysis for RR estimates for bullying victimisation and anxiety disorders are presented in Table 2. Individuals experiencing bullying victimisation including victim-perpetration in childhood and adolescence were found to have twice the risk [RR  =  1.98 (95% CI 1.71–2.30)] of later development of anxiety disorders compared to individuals not involved in bullying. When adjusting for baseline anxiety, the pooled RR was reduced to 1.56 (95% CI 1.32–1.85).

**Table 2.** Relative risk (RR) estimates for bullying victimisation and anxiety disorders from meta-analyses^a^

Adjustment status		Data points	Pooled RR	95% CI Lower bound	95% CI Upper bound	*I*^2^ (%)	Cochran's *Q*	Test for heterogeneity (*p*-value)
Unadjusted	Pooled RR victimisation only	15	1.83	1.41	2.38	67.96	43.70	<0.001
	Pooled RR victimisation including victim-perpetration^b^	17	1.90	1.47	2.46	74.62	63.04	<0.001
	Pooled RR including OR = RR assumption/victimisation including victim-perpetration^b^	19	1.88	1.47	2.41	72.40	65.21	<0.001
Adjusted for demographic, family and other environmental factors^c^	Pooled RR victimisation only	4	1.98	1.70	2.31	5.78	3.18	<0.001
	Pooled RR victimisation including victim-perpetration^b^	5	1.98	1.71	2.30	0	3.18	<0.001
	Pooled RR including OR = RR assumption/victimisation including victim-perpetration^b^	7	1.89	1.67	2.13	0	5.39	<0.001
Adjusted for anxiety at baseline in addition to demographic, family and/or environmental factors	Pooled RR victimisation only	12	1.55	1.29	1.87	59.90	26.12	<0.001
	Pooled RR victimisation including victim-perpetration^b^	14	1.56^d^	1.32	1.85	50.86	26.45	<0.001
	Pooled RR including OR = RR assumption/victimisation including victim-perpetration^b^	20	1.52	1.35	1.72	34.06	28.81	<0.001

a Odds ratios (ORs) for bullying victimisation and anxiety disorders: ORs from original papers converted to RR estimates (Di Pietrantonj, 2006); included studies reported either traditional bullying only, cyberbullying only, traditional bullying and cyberbullying as a single estimate, or traditional bullying and cyberbullying as separate estimates (both estimates included); if studies reported two or more levels of frequency, higher level of frequency included; where studies reported anxiety disorders, general anxiety, social phobia, panic disorders, agoraphobia, anxiety disorder has been chosen as representative estimate of this study.

b Some studies reported estimates for victimisation as well as victim-perpetration, both estimates were included.

c Where studies adjusted for demographic, environmental factors and family factors separately and/or some variables combined, best adjusted estimates were included.

d Pooled RR used for further analyses.

The results of the meta-analysis for RR estimates for bullying victimisation and depressive disorders are presented in Table 3. The pooled RR for depressive disorders for individuals who experienced bullying victimisation (including victim-perpetration) compared to those not involved in bullying was 1.90 (95% CI 1.56–2.32). Those exposed to bullying victimisation including victim-perpetrators had 1.9 times higher risk of later development of depressive disorders. The pooled RRs calculated based on ORs after adjusting for baseline depression was 1.80 (95% CI 1.56–2.08), indicating that those who had been bullied had 1.8 times higher risk of later development of depressive disorders. For both health outcomes, this study pooled RRs with and without OR = RR assumption and there were no significant differences in the RR estimates.

**Table 3.** Relative risk (RR) estimates for bullying victimisation and depressive disorders from meta-analyses^a^

Adjustment status		Data points	Pooled RR	95% CI Lower bound	95% CI Upper bound	*I*^2^ (%)	Cochran's *Q*	Test for heterogeneity (*p*-value)
Unadjusted	Pooled RR victimisation only	18	1.78	1.53	2.09	77.44	75.36	<0.001
	Pooled RR victimisation including victim-perpetration^b^	20	1.85	1.55	2.19	80.68	98.38	<0.001
	Pooled RR including OR = RR assumption/victimisation including victim-perpetration^b^	24	1.84	1.59	2.14	79.16	110.37	<0.001
Adjusted for demographic, family and environmental factors^c^	Pooled RR victimisation only	10	1.89	1.54	2.33	58.26	21.56	<0.001
	Pooled RR victimisation including victim-perpetration^b^	11	1.90	1.56	2.32	55.11	22.28	<0.001
	Pooled RR including OR = RR assumption/victimisation including victim-perpetration^b^	20	1.72	1.38	2.15	75.16	76.51	<0.001
Adjusted for depression at baseline in addition to demographic, family and/or environmental factors	Pooled RR victimisation only	9	1.74	1.51	2.02	0	7.64	<0.001
	Pooled RR victimisation including victim-perpetration^b^	11	1.80^d^	1.56	2.08	0	9.90	<0.001
	Pooled RR including OR = RR assumption/victimisation including victim-perpetration^b^	23	1.73	1.46	2.05	70.62	74.88	<0.001

a Odds ratios (ORs) for bullying victimisation and depressive disorders: ORs from original papers converted to RR estimates (Di Pietrantonj, 2006); included studies reported either traditional bullying only, cyberbullying only, traditional bullying and cyberbullying as a single estimate, or traditional bullying and cyberbullying as separate estimates (both estimates included); if studies reported two or more levels of frequency, higher level of frequency included.

b Some studies reported estimates for victimisation as well as victim-perpetration, both estimates were included.

c Where studies adjusted for demographic, environmental factors and family factors separately and/or some variables combined, best adjusted estimates were included.

d Pooled RR used for further analyses.

### Population attributable fractions and attributable burden

For exposure to bullying victimisation, the calculated PAF for depressive disorders was 10.82% (95% uncertainty interval 5.71–16.05%) and for anxiety disorders was 7.83% (95% uncertainty interval 3.51–12.73%) (Table 4). Overall, bullying victimisation during childhood and adolescence accounted for 0.52% of all DALYs (95% uncertainty interval 0.33–0.72%) for both sexes and all ages (Table 4) in Australia in 2017. For both sexes in the age group 10–24 years, 1.39% of all DALYS in Australia were attributable to bullying victimisation (95% uncertainty interval 0.87–1.90%).

**Table 4.** Estimated burden attributable to bullying victimisation, Australia

DALYs by cause	PAF		DALYs for both sexes and all ages for Australia (GBD 2017)	DALYs attributable to bullying victimisation in Australia for both sexes and all ages (*N*/%)		DALYs for both sexes and ages 10–24 years for Australia (GBD 2017)	DALYs attributable to bullying victimisation in Australia for both sexes and ages 10–24 years (*N*/%)	
Anxiety disorders	7.83%		138 296	10 829		30 877	2418	
95% Uncertainty interval	3.51%	12.73%						
Proportion of total DALYs				0.18%			0.51%	
95% Uncertainty interval				0.08%	0.30%		0.23%	0.83%
Depressive disorders	10.82%		183 205	19 827		38 449	4161	
95% Uncertainty interval	5.71%	16.05%						
Proportion of total DALYs				0.34%			0.88%	
95% Uncertainty interval				0.18%	0.50%		0.46%	1.30%
Anxiety + depressive disorders				30 656			6578	
95% Uncertainty interval				19 304	42 260		4129	9018
All causes			5 868 041			473 825		
Proportion of total DALYs				0.52%			1.39%	
95% Uncertainty interval				0.33%	0.72%		0.87%	1.90%

PAF, population attributable fraction; DALYs, disability-adjusted life years.

GBD 2017  =  source data for the number of DALYs for anxiety and depressive disorders (Kyu *et al*., 2018).

## Discussion

The current study assessed the burden of disease attributable to bullying victimisation during childhood and adolescence in Australia. The systematic review identified 22 longitudinal studies reporting an association between bullying victimisation in childhood and later development of anxiety and depressive disorders. Results showed that bullied children are at a significantly increased risk of later developing anxiety and depressive disorders compared with children not involved in bullying. This association remained statistically significant after controlling for demographic, family and other environmental factors, as well as baseline anxiety and/or depression. This result supports a causal relationship between bullying victimisation and the two outcome variables. Anxiety and depressive disorders have a high prevalence and are significant contributors to the burden of disease. The current study estimated that 7.83% of anxiety disorders and 10.82% of depressive disorders are attributable to exposure to bullying victimisation during childhood and adolescence. It is important to understand not only the prevalence of mental disorders, but also the burden of illness that is attributable to their associated disability. This form of evidence informs the allocation of resources aimed at improving the health outcomes of people with mental disorders. Mental disorders are ranked fourth in Australia in terms of overall DALYs, and anxiety and depressive disorders are the most prevalent mental illnesses (Kyu *et al*., 2018). An estimated 30 656 DALYs (95% uncertainty interval 19 304– 42 260) or 0.52% of DALYs for all causes in both sexes and all ages; and 6578 DALYs (95% uncertainty interval 4129–9018) or 1.39% of DALYs for all causes in both sexes in the age group 10–24 years in Australia were attributable to bullying victimisation during childhood and adolescence.

Recently, GBD 2017 comparative risk assessment added bullying victimisation as a risk factor for anxiety and depressive disorders (Stanaway *et al*., 2018). The methodology used in GBD 2017 combined anxiety and depressive disorders data into a single estimate that pooled the RRs for both disorders together [RR  =  1.79 (95% CI 1.63–1.98)]. Although a different type of meta-analytic method was used, this estimate is consistent with estimated RRs for those health outcomes in this study [anxiety disorders RR  =  1.56 (95% CI 1.32–1.85) and depressive disorders RR  =  1.80 (95% CI 1.56–2.08)]. Furthermore, the global study used adjusted prevalence estimates and reported results for specific age groups. The current study used the pooled prevalence of bullying victimisation and reports attributable DALYs across all age groups and for ages 10–24 years. The overall estimates of attributable DALYs due to bullying victimisation is higher (1.39%) for ages 10–24 years compared to other age groups – a result consistent with GBD 2017. Although these studies reported the burden attributable to bullying victimisation in different ways, they are broadly consistent in finding that bullying victimisation makes a significant contribution to DALYs.

It has been proposed that a reduction in the population prevalence of mental disorders in Australia and other high-income countries could be achieved through a systematic effort to prevent bullying victimisation (Scott *et al*., 2014). A variety of effective intervention programmes have been implemented to address bullying in many countries. A systematic review and meta-analysis evaluating school-based anti-bullying programmes reported that interventions can reduce bullying victimisation by 15–16% and bullying perpetration by 19–20% (Gaffney *et al*., 2018*b*). Programmes to specifically address cyberbullying have also been developed, and are reported to reduce cyberbullying victimisation by 14% and cyberbullying perpetration by 10–15% (Gaffney *et al*., 2018*a*). Using results from this study, a reduction of between 10 and 20% in the prevalence of bullying victimisation among children and adolescents would result in the avoidance of 3000–5000 DALYs due to anxiety and depressive disorders in both sexes and all ages.

The current study illustrates the potential health benefits that could arise from the implementation of programmes to reduce bullying victimisation in Australia. To further support the case for implementation of bullying prevention, there is a need to quantify the costs related to anxiety and depressive disorders associated with bullying victimisation, as well as the value of lost productivity due to consequences of exposure to bullying victimisation during childhood and adolescence.

### Strengths and limitations

There are several strengths of this study. The pooled findings from longitudinal cohort studies provide the opportunity to avoid recall bias of bullying victimisation. Also, the quality effects model allows quantifying studies not only according to sample size but also by study quality, giving greater weight to studies of high quality. Furthermore, this study controlled for pre-existing mental health problems by using pooled RRs adjusted for baseline mental health outcomes in order to quantify PAFs. Otherwise, the results would be an overestimate of the burden because the continuation of pre-existing psychopathology would not have been accounted for (Moore *et al*., 2014). Finally, PAF estimates provide an opportunity to quantify the burden of mental disorders that could be avoided in future by reducing bullying victimisation prevalence through anti-bullying interventions.

The current study also had limitations. Due to the limited number of studies, the RR estimates for bullying victimisation and mental disorders were derived from research where the bullying victimisation was reported from different sources (self-reported, teacher and/or parent reported), while the prevalence estimate of bullying victimisation experience was from meta-analyses which were derived only from studies where bullying victimisation was self-reported. In addition, there was a large variance in the follow-up period of included longitudinal cohort studies. The influence of this variation has not been examined. For some included studies, both the exposure *and* the outcome occurred within the period of childhood and adolescence (i.e. 18 years or younger). In addition, there is a waning effect on outcomes with effect sizes that likely diminish over time (Stanaway *et al*., 2018). Hence, applying PAFs based on current prevalence in childhood and adolescence and a single RR value to the burden of anxiety and depressive disorders across all ages may overestimate the overall attributable burden. Finally, the focus of this study was on anxiety and depressive disorders only. But there are also other consequences of bullying victimisation including poor general health, non-suicidal self-injury and substance use, which were not included (Moore *et al*., 2017). However, the evidence-base for a causal relationship for many of these outcomes is limited and no firm conclusions have yet been made.

## Conclusion

The quantification of the disease burden attributable to bullying victimisation demonstrates the significant morbidity caused by this exposure during childhood and adolescence. For this reason, the prevention of bullying victimisation should be a priority for public health policy and action. Health and education systems need to respond by implementing evidence-based intervention programmes that reduce bullying in schools. The provision of a more preventive approach has the potential to reduce the burden of disease and improve the mental health of Australians.

## Availability of Data and Materials

The datasets used and analysed during the systematic review and meta-analyses are available from the corresponding author on request.

## Appendix 2: Review protocol

1. Previous systematic review was conducted using PubMed, EMBASE, ERIC and PsycINFO electronic databases from inception until 28 February 2015 and included longitudinal and cross-sectional studies that examined the association between health and psychological outcome and bullying victimisation (Moore *et al*., 2017).
2. Update this systematic review from 1 January 2015 until 18 August 2018 and include longitudinal studies only.

**Primary database:** Four electronic databases (PubMed, EMBASE, ERIC and PsycINFO)

**Search terms:**

**Table:**

Database	Search group	Search terms
Embase	Bullying victims	(bullied OR ‘bullying’/exp OR bullying OR teas* OR harass* OR victimization OR victimisation OR intimidat*) AND (child* OR adolescen*) AND (outcome OR harm OR consequences OR ‘risk’/exp OR risk) AND (‘depress*’:ab,ti OR ‘anxiety’:ab,ti) AND (‘longitudinal’:ab,ti OR ‘cohort’:ab,ti) AND [2015-2018]/py 99
PubMed	Bullying victims	((((((bullied OR bullying OR teas* OR harass* OR victimization OR victimisation OR intimidat*) AND (child* OR adolescen*) AND (outcome OR harm OR consequences OR risk))) AND (depress* OR anxiety)) AND (‘2015/01/01’[PDat] : ‘3000/12/31’[PDat]) AND Humans[Mesh])) AND (longitudinal[Title/Abstract] OR cohort[Title/Abstract]) 111
ERIC	Bullying victims	(((Keywords:bullied OR Keywords:bullying OR Keywords:teas* OR Keywords:harass* OR Keywords:victimization OR Keywords:victimisation OR Keywords:intimidat*) AND (Keywords:child* OR Keywords:adolescen*) AND (Keywords:outcome OR Keywords:harm OR Keywords:consequences OR Keywords:risk)), and Publication Type: ‘Journal Articles’) AND (longitudinal OR cohort) AND (depress* OR anxiety) Limiters – Published Date: 20150101–20181231 77
PsycINFO	Bullying victims	((Bullying OR bullied OR teas* OR harass* OR victimization OR victimisation OR intimidat*) AND (child* OR adolescen*) AND (outcome OR harm OR consequences OR risk)) AND AB (depress* OR anxiety) AND AB (longitudinal OR cohort) Limiters: Publication year: 2015–2018 115

**Additional searching:**

- Reference list review (any article pulled for possible inclusion)
- Contact with study authors
- Any article deemed suitable by reviewers is included for closer examination.

### Inclusion/exclusion criteria

**Inclusion criteria:**

Studies were included if they were published in a peer-reviewed journal, reported an association between exposure to bullying victimisation and anxiety disorders or depressive disorders and were population based.

1. ***Question of interest*:** Are individuals who have experienced bullying victimisation in childhood and adolescence at an increased risk of later development of anxiety disorders and depressive disorders compared with those who are not exposed?
  ***Population*:** General population, children adolescents or adults.
    ***Exposure***: Victims of bullying – exposure to negative actions repeatedly and over time from one or more people and involves a power imbalance between the perpetrator/s and the victim.
    ***Exposure measurement***: Bullying victimisation could be self-reported, teacher reported, parent reported or clinician reported on either a validated scale or a questionnaire designed specifically for that study.
    ***Age range for exposure***: Bullying victimisation occurred between 0 and 18 years but studies also included if age not reported.
    ***Comparison***: Individuals not exposed to bullying victimisation.
    ***Outcome***: Two main health consequences of bullying: anxiety disorders and depressive disorders.
    ***Outcome measurement***: Diagnosed by a health professional or an objective measure, standardised/non-standardised screening instrument or self-reported outcomes also accepted.
2. ***Study designs of interest*:** Prospective and retrospective cohort
  No limits on language. Published since January 2015 up to 18 August 2018.
    Articles in languages other than English deemed relevant based on its abstract are translated.

**Exclusion criteria:**

Articles initially excluded if they are duplicates or if the title clearly demonstrates that the exposure and outcome of interest are not the focus of the article. Articles are then excluded based on the following:

- The article does not examine an association between bullying victimisation and depression or anxiety (7).
- The study used cross-sectional data. Subsequently, one paper based on a longitudinal study was excluded because analyses were based on data within one wave, making them essentially cross-sectional in character (3).
- No effect size and uncertainty information reported or cannot be computed from information given (22).
- Bullying is considered as a risk factor/mediator between two other exposure and outcome variables.
- The study investigated the promotive and protective role of environmental, social and family support on the longitudinal relationship between victimisation and health outcomes (1).
- There is no control group or comparison group (just looked at the characteristics of the exposed group).
- The study was not population based.
- The study is a review article, a letter to the editor or a published abstract from a conference.
- The study based on unique population such as youth with disabilities, HIV/AIDS affected children and adolescents, bisexual and lesbian women, adults born at extremely low birth weight (4).
- Where there were multiple papers that reported on the same study population, the study that reported more detailed information was included (2).
- Studies used a dimensional peer nomination indicator (1).
- Studies examined mental and emotional wellbeing predictors of bullying victimisation (1).
- Studies examined bullying victimisation and health outcome at preschool age (1).

**Data abstraction form**

**Identification of the study:**

1. Record the first authors' last name, initials
2. Record the journal name
3. Record the year of publication
4. Record the volume number
5. Record the page numbers

**Characteristics of the study:**

1. Study period
2. Study design
3. Sample size and gender
4. Retrospective/prospective analysis
5. Country
6. Type of bullying, frequency of bullying
7. Assessment of exposure
8. Outcomes (depression or anxiety)
9. Assessment of outcome

**Other data:**

1. Effect size and 95% confidence interval: converted to relative risk (RR) estimates by Di Pietrantonj's (2006) method.

***Quality assessment:*** Quality of studies was assessed using the tool above which was adapted from a tool for assessing the risk of bias in cohort studies (Newcastle–Ottawa scale for cohort studies) (Wells et al., 2000). The total quality score for each study is the sum of the scores for individual assessment items, the maximum quality score for this study was 11. This is converted to a proportional quality score for use in Meta-XL version 5.3 (the total quality score divided by the maximum score possible).

**Table:** **Quality assessment tool:**

Quality criteria		Quality score
*Selection*		
1.	Study design	Prospective cohort = 1 Retrospective cohort = 0
2.	Representativeness of the population	Representativeness of the wider population: Population-based representative/clear description by authors that study sample is representative of the wider population = 1 No description of sample/inadequate description/targeted study or sample not representative (i.e. based on boys only or girls only) = 0
3.	Selection of the non-exposed cohort/controls	Drawn from the same population = 1 Drawn from a different source/no description = 0
4.	Definition of bullying provided for the participants	Yes = 1 No/no description = 0
5.	Ascertainment of exposure to bullying: How the exposure to bullying was measured?	a. Was bullying measured/operationalised according to frequency (as opposed to a yes/no response)? b. Was prevalence estimated using a threshold that meets the criteria of repetition (threshold greater than ‘once or twice’)? Responses coded: yes = 1 (if yes to both questions) Partial = 0.5 (if yes to one question) No = 0 (if no to both questions)
*Comparability*		
6.	Appropriate methods to control confounding:	Controlled for prior psychological problems or outcome measure at baseline only/controlled for prior psychological problems or outcome measure at baseline and demographic or SES or environmental and family factors = 2 Controlled for demographic + SES or environmental and family factors only = 1 Controlled for demographic factors only or there was no confounding controlled for = 0
Outcome		
7.	Ascertainment of outcome: How was the outcome measured?	Clinician reported or objective measure [use of a structured diagnostic interview for DSM-III/IV (DIS, DISC, CIDI) (mental health)] = 1 Questions from published health surveys/screening instruments or own system /symptoms described/no system/not specified/self-reported = 0
8.	Adequacy of follow-up of cohorts	Completeness good (⩾80%), with description of those lost to follow-up = 1 Completeness poor (<80%) or no statement = 0
9.	Was follow-up long enough for depression and anxiety to occur	More than 6 months = 1 Less than 6 months = 0
10.	Appropriate statistical analysis and information provided	Exposed/non-exposed case numbers reported = 1 Exposed/non-exposed case numbers not reported = 0

## Appendix 3

**Table A1.** Summary of study characteristics

	First author/publication year	Setting	Sample source	Gender	Type of exposure	Age of exposure (year)	Ascertainment of exposure	Health outcome	Age of outcomes assessed (years)	Assessment of health outcome
1	Bowes *et al*. (2015)	Avon, UK, Europe	Avon Longitudinal Study of Parents and Children (ALSPAC)	Males and females	Bullying victimisation (frequent and sometimes)	8,10,13	A modified version of the bullying and friendship interview (self-reported)	Depression	18	A self-administered computerised version of the clinical interview schedule-revised CIS-R
2	Copeland *et al*. (2013)	11 counties in Western North Carolina, USA, North America	The Great Smoky Mountain Study (GSMS)	Males and females	Bullying victimisation and bullying victim-perpetration	9–16	The child and their parent reported on whether the child had been bullied or teased or bullied others [part of Child and Adolescent Psychiatric Assessment (CAPA)]	Anxiety disorders, general anxiety, panic disorder, agoraphobia and depressive disorders: major/minor depression, and dysthymia	19, 21, 24–26	The Young Adult Psychiatric Assessment (YAPA) – structured diagnostic interview-diagnoses made included any DSM-IY anxiety disorders and depressive disorders
3	Fahy *et al*. (2016)	East London, UK, Europe	The Olympic Regeneration in East London (ORiEL) study	Males and females	Cyberbullying victimisation and cyberbullying victim-perpetration	11–12	A six-item scale (self-reported)	Depressive symptoms and social anxiety symptoms	12–14	Short Mood and Feelings Questionnaire (SMFQ)
4	Farrington *et al*. (2011)	PA, USA, North America	The Pittsburgh Youth Study	Males	Bullying victimisation	10–14	A specific questionnaire on bullying was completed by the boy and his mother	Depression	11–16	The boys completed the Recent Mood and Feelings Questionnaire and the mothers and teachers completed the child behaviour checklist (CBCL)
5	Fekkes *et al*. (2006)	The Netherland, Europe	The study population was derived from 18 Dutch elementary schools	Males and females	Bullying victimisation	9–11	The Dutch version of the Olweus Bully/Victim Questionnaire (self-reported)	Anxiety and depression	10–12	KIVPA, a Dutch instrument to measure psychosocial problems among children
6	Geoffroy *et al*. (2018)	Quebec, Canada, North America	The Quebec Longitudinal Study of Child Development	Males and females	Physical, verbal, relational and cyber bullying victimisation (moderate and severe)	7–13	A modified version of the Self-Report Victimization Scale	Generalised anxiety problems, social anxiety problems and depression/dysthymia problems	15	The Mental Health and Social In-adaptation Assessment
7	Hemphill *et al*. (2011)	Victoria, Australia and Washington State, USA, North America	The International Youth Development Study (IYDS)	Males and females	Bullying victimisation	Year 7 and year 10	A modified version of the Communities that Care: bullying victimisation was assessed by asking students if they had been ‘bullied recently’ (teased or called names, had rumours spread about you, been deliberately left out of things, threatened physically or actually hurt) (self-reported)	Depressive symptoms	Year 11	The self-report Short Mood and Feelings Questionnaire (SMFQ)
8	Hemphill *et al*. (2014)	Victoria, Australia	The sample for this study comprised Victorian students from the International Youth Development Study (IYDS)	Males and females	Bullying victimisation	16–17	A modified version of the Communities that Care: bullying victimisation was assessed by asking students if they had been ‘bullied recently’ (teased or called names, had rumours spread about you, been deliberately left out of things, threatened physically or actually hurt) (self-reported)	Depressive symptoms	18–19	Depressive symptoms were measured using the Kessler Psychology Distress Scale
9	Hemphill *et al*. (2015)	Victoria, Australia and Washington State, USA, North America	The International Youth Development Study (IYDS)	Males and females	Cyberbullying victimisation and cyberbullying victim-perpetration	14–16.5	Global single question: been bullied by another student who has used technology such as mobile-phones, the Internet, computers, answering machines or cameras? (self-reported)	Depressive symptoms	16–18.5	Depressive symptoms were measured using the self-report Short Mood and Feelings Questionnaire
10	Kaltiala-Heino *et al*. (2010)	Tampere and Vantaa, Finland, Europe	The Adolescent Mental Health Cohort Study (AMHC)	Males and females	Bullying victimisation	15	Question derived from the WHO Youth Health Study: the respondents were asked how frequently they had been bullied during the ongoing school term (self-reported)	Depression	17	R-BDI, a Finnish modification of the 13-item Beck Depression Inventory
11	Klomek *et al*. (2008)	Finland, Europe	From a Boy to a Man Study	Males	Bullying victimisation (frequent and sometimes)	8	The child himself/herself, a parent, and a teacher were asked about being victims of bullying	Depression symptoms (mild and severe)	18	The Beck's Depression Inventory (BDI)
12	Lereya *et al*. (2015)	Avon, South West England, UK, North Carolina, USA, Europe and North America	The Avon Longitudinal Study of Parents and Children in the UK (ALSPAC) and the Great Smoky Mountains Study in the USA (GSMS) longitudinal studies	Males and females	Bullying victimisation (being bullied only refers to being bullied by peers in at least one time point)	ALSPAC: 8–13; GSMS: 9–16	ALSPAC: child interviewed: Bullying and Friendship Interview Schedule; GSMS: the child and their parent reported on whether the child had been bullied or teased or bullied others [part of Child and Adolescent Psychiatric Assessment (CAPA)]	ALSPAC: anxiety (generalised anxiety disorder, social phobia, specific phobia, panic disorder or agoraphobia); GSMS: anxiety disorder (generalised anxiety, agoraphobia, panic disorder, social phobia, obsessive–compulsive disorder and post-traumatic stress disorder)	ALSPAC :18; GSMS: 19, 21,24–26	ALSPAC: a reliable and validated self-administered computerised version of the Clinical Interview Schedule (CIS-R); GSMS: Young Adult Psychiatric Assessment (YAPA)
13	Patton *et al*. (2008)	Washington (WA), USA, and Victoria (VIC), Australia	The International Youth Development Study (IYDS)	Females	Bullying victimisation	10–15 (annually)	Self-reported global single question: Have you been bullied recently (teased or called names, had rumours spread about you, been deliberately left out of things, threatened physically or actually hurt)?	High depressive symptoms (12 months later)	10–15 (annually)	The Short Mood and Feelings Questionnaire designed for epidemiological survey research with adolescents. The onset of new depressive symptoms in the female subjects
14	Ranta *et al*. (2013)	Finland, Europe	The Adolescent Mental Health Cohort Study (AMHCS)	Males and females	Direct bullying victimisation and relational bullying victimisation	15	The self-reported question assessing subjection to bullying was derived from a WHO youth health study: ‘How frequently have you been bullied during the ongoing school term?’ Relational victimisation was assessed with a question: ‘How frequently have other pupils not wanted to be with you and you had to be by yourself during the ongoing school term?’	Social phobia	17	Social phobia was assessed with the Social Phobia Inventory (SPIN): a 17-item self-report questionnaire for measuring fear, avoidance behaviours and physiological arousal in performance or social situations
15	Rothon *et al*. (2011)	London, UK, Europe	The Research with East London Adolescents: Community Health Survey (RELACHS)	Males and females	Bullying victimisation	11–14	Self-reported questions: ‘How often have you been bullied in school this term?’ A further category of ‘never bullied’ was added based on another item: ‘Have you ever been bullied at school?’	Depressive symptoms	13–16	The Short Moods and Feelings Questionnaire (SMFQ)
16	Schoon and Montgomery (1997)	UK, Europe	The National Child Development Study (NCDS)	Males and females	Bullying victimisation (frequent and sometimes)	Birth to 7	The parents were asked to indicate whether the description is ‘often’, ‘sometimes’ or ‘never’ applies. Description: ‘The child is harassed by other children’	Depression	33	To assess emotional distress and somatic symptoms associated with a depressive state, Ruter's Malaise questionnaire was used
17	Silberg *et al*. (2016)	Virginia, USA, North America	The Virginia Twin Study of Adolescent Behavioural Development (VTSABD) and The Young Adult Follow-Up Study (YAFU)	Males and females	Bullying victimisation	8–17	Self-reported and mother reported (CAPA) assessment of bullying victimisation has been used	Major depressive episode, generalised anxiety and panic attacks	≥18	The DSM-III-R based Structured Clinical Interview (SCID)
18	Sourander *et al*. (2007)	Finland, Europe	From a Boy to a Man	Males	Bullying victimisation	8	The child himself/herself, a parent, and a teacher were asked about being victims of bullying	Depressive disorders and anxiety disorders	18–23	The ICD-10 psychiatric diagnoses were based on health examinations performed by general physicians or senior psychiatrists
19	Sourander *et al*. (2016)	Finland, Europe	Finnish Nationwide 1981 Birth Cohort Study	Males and females	Bullying victimisation and bullying victim-perpetration (frequent)	8	Child, teacher, and parent were asked about bullying victimisation	Depressive disorders (ICD-10 codes F32-F39); anxiety, stress-related, adjustment, and somatoform disorders (ICD-10 codes F40-F48; abbreviated anxiety)	16–29	Use of specialised services for psychiatric disorders from 16 to 29 years of age was obtained from a nationwide hospital register, including outpatient and inpatient treatment
20	Stapinski *et al*. (2014)	Avon, UK, Europe	The Avon Longitudinal Study of Parents and Children (ALSPAC)	Males and females	Bullying victimisation (frequent and occasional)	13	A modified version of the Bullying and Friendship Interview Schedule (self-reported)	Any depression diagnosis, any anxiety disorders, general anxiety disorders, social phobia, specific phobia, panic disorder and agoraphobia	18	A self-administered computerised version of the CIS-R
21	Takizawa *et al*. (2014)	England, Scotland and Wales, Europe	The British National Child Development Study (NCDS)	Males and females	Bullying victimisation (frequent and occasional)	7 and 11	Parents were interviewed when participants were 7 and 11 years old	Any depression and any anxiety disorder	45	The depression and anxiety modules of the Revised Clinical Interview Schedule, administered by trained research nurses using computer-assisted personal interviewing as part of a clinical examination in the participants’ homes
22	Zwierzynska *et al*. (2013)	Avon, UK, Europe	Avon Longitudinal Study of Parents and Children (ALSPAC)	Males and females	Bullying victimisation (stable and unstable)	8 and 10	Child reports were derived from a modified version of the Bullying and Friendship Interview Schedule at 8 and 10 years. Mother and teacher reports were derived from a single item ‘Child is picked on or bullied by other children’ at 7, 8 and 9 years from the mothers, and at 7 and 10 years from the teachers	Any anxiety disorder diagnosis and major depression diagnosis at 13 years, early (at 11–12 years) and late depression symptoms (at 13–14 years)	11–14	The Short Mood and Feelings Questionnaire at ages 11, 12, 13 and 14 years; depressive disorder and anxiety disorder at 13 years measured by the Development and Well-Being Assessment

**Table A2.** Quality assessment

	Total score (maximum 11)	Study design: Prospective cohort = 1 Retrospective cohort = 0	Representativeness of the wider population: Population-based representative/clear description by authors that study sample is representative of the wider population = 1 No description of sample/inadequate description/targeted study or sample not representative (i.e. based on boys only or girls only) = 0	Selection of the non-exposed cohort/controls: Drawn from the same population = 1 Drawn from a different source/no description = 0	Definition of bullying provided for the participants: Yes = 1 No/no description = 0	Ascertainment of exposure to bullying: How the exposure to bullying was measured? Responses coded: Yes = 1 (if yes to both questions) Partial = 0.5 (if yes to one question) No = 0 (if no to both questions)			Appropriate methods to control confounding: Controlled for prior psychological problems or outcome measure at baseline only/controlled for prior psychological problems or outcome measure at baseline and demographic or SES or environmental and family factors = 2 Controlled for demographic + SES or environmental and family factors only = 1 Controlled for demographic factors only/SES only/environmental and family factor only/there was no confounding controlled for/no statement = 0	Ascertainment of outcome: How was the outcome measured? Clinician reported or objective measure [use of a structured diagnostic interview for DSM-III/IV (DIS, DISC, CIDI) (mental health)] = 1 Questions from published health surveys/screening instruments or own system /symptoms described/no system/not specified/self-reported = 0	Adequacy of follow-up of cohorts Completeness good (⩾80%), with description of those lost to follow-up = 1 Completeness poor (<80%) or no statement = 0	Was follow-up long enough for depression and anxiety to occur More than 6 months = 1 Less than 6 months = 0	Appropriate statistical analysis and information provided Exposed/non-exposed case numbers reported = 1 Exposed/non-exposed case numbers not reported = 0
						a/ Was bullying measured/operationalised according to frequency (as opposed to a yes/no response)?	b/ Was prevalence estimated using a threshold that meets the criteria of repetition (threshold greater than ‘once or twice’)?	Overall					
Bowes *et al*. (2015)	7^a^	1	1	1	0	Yes	Yes	1	0	1	0	1	1
	8^b^	1	1	1	0	Yes	Yes	1	1	1	0	1	1
	9^c^	1	1	1	0	Yes	Yes	1	2	1	0	1	1
Copeland *et al*. (2013)	8^a^	1	1	1	1	No	No	0	0	1	1	1	1
	10^a^	1	1	1	1	No	No	0	2	1	1	1	1
Fahy *et al*. (2016)	4.5^a^	1	1	1	0	Yes	No	0.5	0	0	0	1	0
	5.5^b^	1	1	1	0	Yes	No	0.5	1	0	0	1	0
	6.5^c^	1	1	1	0	Yes	No	0.5	2	0	0	1	0
Farrington *et al*. (2011)	6^a^	1	0	1	0	No description	No description	0	0	1	1	1	1
	7^b^	1	0	1	0	No description	No description	0	1	1	1	1	1
Fekkes *et al*. (2006)	6^a^	1	0	1	1	Yes	Yes	1	0	0	0	1	1
	7^b^	1	0	1	1	Yes	Yes	1	1	0	0	1	1
Geoffroy *et al*. (2018)	5.5^a^	1	1	1	0	Yes	No	0.5	0	0	0	1	1
	6.5^b^	1	1	1	0	Yes	No	0.5	1	0	0	1	1
	7.5^c^	1	1	1	0	Yes	No	0.5	2	0	0	1	1
Hemphill *et al*. (2011)	5^a^	1	0	1	0	Yes	Yes	1	0	0	1	1	0
	7^c^	1	0	1	0	Yes	Yes	1	2	0	1	1	0
Hemphill *et al*. (2014)	6^a^	1	0	1	0	Yes	Yes	1	0	0	1	1	1
	7^b^	1	0	1	0	Yes	Yes	1	1	0	1	1	1
Hemphill *et al*. (2015)	6^b^	1	0	1	0	Yes	Yes	1	1	0	1	1	0
Kaltiala-Heino *et al*. (2010)	6^a^	1	0	1	1	Yes	Yes	1	0	0	0	1	1
	7^b^	1	0	1	1	Yes	Yes	1	1	0	0	1	1
	8^c^	1	0	1	1	Yes	Yes	1	2	0	0	1	1
Klomek *et al*. (2008)	6.5^a^	1	0	1	0	Yes	No	0.5	0	1	1	1	1
	8.5^c^	1	0	1	0	Yes	No	0.5	2	1	1	1	1
Lereya *et al*. (2015)	6.5^a^	1	1	1	0	Yes	No	0.5	0	1	0	1	1
	7.5^b^	1	1	1	0	Yes	No	0.5	1	1	0	1	1
	7.5^a^	1	1	1	0	Yes	No	0.5	0	1	1	1	1
	8.5^b^	1	1	1	0	Yes	No	0.5	1	1	1	1	1
Patton *et al*. (2008)	6^b^	1	0	0	0	Yes	Yes	1	1	1	1	1	0
	7^c^	1	0	0	0	Yes	Yes	1	2	1	1	1	0
Ranta *et al*. (2013)	6^a^	1	0	1	1	Yes	Yes	1	0	0	0	1	1
	8^c^	1	0	1	1	Yes	Yes	1	2	0	0	1	1
Rothon *et al*. (2011)	5^b^	1	0	1	0	Yes	Yes	1	1	0	0	1	0
	6^c^	1	0	1	0	Yes	Yes	1	2	0	0	1	0
Schoon and Montgomery (1997)	6.5^a^	1	1	1	0	Yes	No	0.5	0	0	1	1	1
Silberg *et al*. (2016)	5^a^	1	0	1	0	No description	No description	0	0	1	1	1	0
Sourander *et al*. (2007)	6.5^a^	1	0	1	0	Yes	No	0.5	0	1	1	1	1
	8.5^c^	1	0	1	0	Yes	No	0.5	2	1	1	1	1
Sourander *et al*. (2016)	7.5^a^	1	1	1	0	Yes	No	0.5	0	1	1	1	1
	8.5^b^	1	1	1	0	Yes	No	0.5	1	1	1	1	1
	9.5^c^	1	1	1	0	Yes	No	0.5	2	1	1	1	1
Stapinski *et al*. (2014)	6.5^a^	1	1	1	0	Yes	No	0.5	0	1	0	1	1
	8.5^c^	1	1	1	0	Yes	No	0.5	2	1	0	1	1
Takizawa *et al*. (2014)	8.5^c^	1	1	1	0	Yes	No	0.5	2	1	0	1	1
Zwierzynska *et al*. (2013)	6.5^c^	1	1	1	0	Yes	No	0.5	2	0	0	1	0
	7.5^c^	1	1	1	0	Yes	No	0.5	2	1	0	1	0

a There was no confounding controlled for/no statement.

b Controlled for demographic factors only/SES only/environmental and family factor only/demographic  +  SES or environmental and family factors only.

c Controlled for prior psychological problems or outcome measure at baseline only/controlled for prior psychological problems or outcome measure at baseline and demographic or SES or environmental and family factors.

## Appendix 4

See Figs 1–3.
